# Cost-effectiveness of a direct to beneficiary mobile communication programme in improving reproductive and child health outcomes in India

**DOI:** 10.1136/bmjgh-2022-009553

**Published:** 2023-03-23

**Authors:** Amnesty Elizabeth LeFevre, Jai Mendiratta, Youngji Jo, Sara Chamberlain, Osama Ummer, Molly Miller, Kerry Scott, Neha Shah, Arpita Chakraborty, Anna Godfrey, Priyanka Dutt, Diwakar Mohan

**Affiliations:** 1 Division of Public Health Medicine, University of Cape Town, School of Public Health, Cape Town, Western Cape, South Africa; 2 BBC Media Action-India, Delhi, India; 3 Department of Public Health Sciences, School of Medicine, University of Connecticut, Farmington, Connecticut, USA; 4 Independent Consultant, Digital Health & Gender, Delhi, India; 5 Oxford Policy Management, New Delhi, India; 6 International Health, Johns Hopkins University Bloomberg School of Public Health, Baltimore, Maryland, USA; 7 Research & Evidence, Oxford Policy Management, India, New Delhi, Delhi, India; 8 BBC Media Action, London, UK; 9 GivingTuesday India Hub, Delhi, India

**Keywords:** Health economics, Maternal health, Randomised control trial, Prevention strategies, Child health

## Abstract

**Introduction:**

Kilkari is the largest maternal messaging programme of its kind globally. Between its initiation in 2012 in Bihar and its transition to the government in 2019, Kilkari was scaled to 13 states across India and reached over 10 million new and expectant mothers and their families. This study aims to determine the cost-effectiveness of exposure to Kilkari as compared with no exposure across 13 states in India.

**Methods:**

The study was conducted from a programme perspective using an analytic time horizon aligned with national scale-up efforts from December 2014 to April 2019. Economic costs were derived from the financial records of implementing partners. Data on incremental changes in the practice of reproductive maternal newborn and child health (RMNCH) outcomes were drawn from an individually randomised controlled trial in Madhya Pradesh and inputted into the Lives Saved Tool to yield estimates of maternal and child lives saved. One-way and probabilistic sensitivity analyses were carried out to assess uncertainty.

**Results:**

Inflation adjusted programme costs were US$8.4 million for the period of December 2014–April 2019, corresponding to an average cost of US$264 298 per year of implementation in each state. An estimated 13 842 lives were saved across 13 states, 96% among children and 4% among mothers. The cost per life saved ranged by year of implementation and with the addition of new states from US$392 ($385–$393) to US$953 ($889–$1092). Key drivers included call costs and incremental changes in coverage for key RMNCH practices.

**Conclusion:**

Kilkari is highly cost-effective using a threshold of India’s national gross domestic product of US$1998. Study findings provide important evidence on the cost-effectiveness of a national maternal messaging programme in India.

**Trial registration:**

NCT03576157.

**What is already known on this topic**

- There is a paucity of evidence on the value for money of digital health programmes, including direct to beneficiary solutions which provide mobile health information content directly to pregnant and postpartum women.

**What this study adds**

- This study suggests that the Kilkari programme saved an estimated 13 842 lives across 13 states from December 2014 to April 2019 at a total programme cost of US$8.4 million.
- The cost per live saved from US$392 ($385–$393) to $953 ($889–1092) well under the gross domestic product of US$1998 for India. Key drivers included call costs and incremental changes in coverage for key reproductive maternal newborn and child health practices.

**How this study might affect research, practice or policy**

- This is the first study of its kind to demonstrate the cost-effectiveness of a direct to beneficiary mobile health programme being implemented at scale, under real-world conditions.

## Introduction

Direct to beneficiary mobile health services which send health information to new and expectant mothers are among the few types of digital health programmes to have scaled widely in a range of countries globally. At least four programmes globally have scaled to reach over a million subscribers including Aponjon in Bangladesh,^1 2^ mMitra^3^ and Kilkari in India^4^ and MomConnect in South Africa.^5^ While evidence on the impact of these programmes is emerging,^3 4 6^ the limited available data on the value for money and affordability remains a critical impediment to transitioning from donor to government funding and enabling longer term sustainability.^7^

Kilkari is an outbound service that makes weekly, stage-based, prerecorded calls about reproductive, maternal, neonatal and child health directly to families’ mobile phones, starting from the second trimester of pregnancy and until the child is 1-year old. Between its inception in 2012 in Bihar, and its transition to the governmet in April 2019, Kilkari was scaled to 10 million subscribers in over 13 states across India. Current estimates of programmatic reach suggest that the programme has reached over 29 million women and their families across 18 states and currently has 2.5 million active users.^8^ Emerging evidence on the impact of Kilkari suggests that exposure to health information messages may increase immunisation coverage at 10 weeks and lead to shift in contraceptive methods—increasing the use of modern reversible contraceptive methods overall and slightly decreasing the proportion of men or women sterilised since the birth of the child.^9^ Evidence on the cost-effectiveness of the Kilkari programme, however, remains outstanding.

Evidence reviews of the cost-effectiveness and cost-utility of digital health solutions is emerging for mobile health solutions which target older adults,^10^ behaviour change communication apps,^11^ telemedicine in Asia^12^ and mHealth solutions more broadly.^13^ However, less is known about the value for money of direct to beneficiary mobile health programmes operating at scale.^14^ While these services have been implemented widely in a range of settings, the rapid pace of their scale-up has occurred without robust evidence generation on their impact or value for money.^15^ A model-based analysis of the Mobile Alliance for Maternal Action (MAMA) project in South Africa sought to forecast the costs and consequences of scaling-up the text-based delivery of health information messages to pregnant and postpartum women across the province of Gauteng in South Africa.^14^ An earlier study sought to explore the cost-effectiveness of Mobile Technology for Community Health (MOTECH) in Ghana—an interactive voice response (IVR) service similar to Kilkari but inclusive of an additional facility-based data capture application for frontline health workers.^16^ These studies have collectively suggested that direct to beneficiary mobile health information messages may be cost-effective; however, limitations in the underlying study design (none was conducted as part of randomised controlled trials (RCTs)) coupled with programmatic variations and differences in the scale of implementation, limits comparability and syntheses.

The goal of this study is to bolster evidence on the value for money of direct to beneficiary mobile health interventions in low-income and middle-income countries where the majority of maternal and child deaths occur each year. This study aims to determine the cost-effectiveness of exposure to Kilkari—one of the world’s largest direct to beneficiary mobile health service—as compared with the status quo of no mobile health information messages across 13 states in India. We start by presenting programme costs associated with the gradual start-up and implementation of programme activities by state. We next present modelled estimates of lives saved based on data drawn from an individually RCT in the central Indian state of Madhya Pradesh.^17^ Finally, we present estimates of cost-effectiveness and findings from uncertainty analyses. This is the first study of its kind to explore the value for money of a direct to beneficiary mobile health programme at scale under real-world conditions of implementation.

## Methods

### Study setting

**Figure 1:**
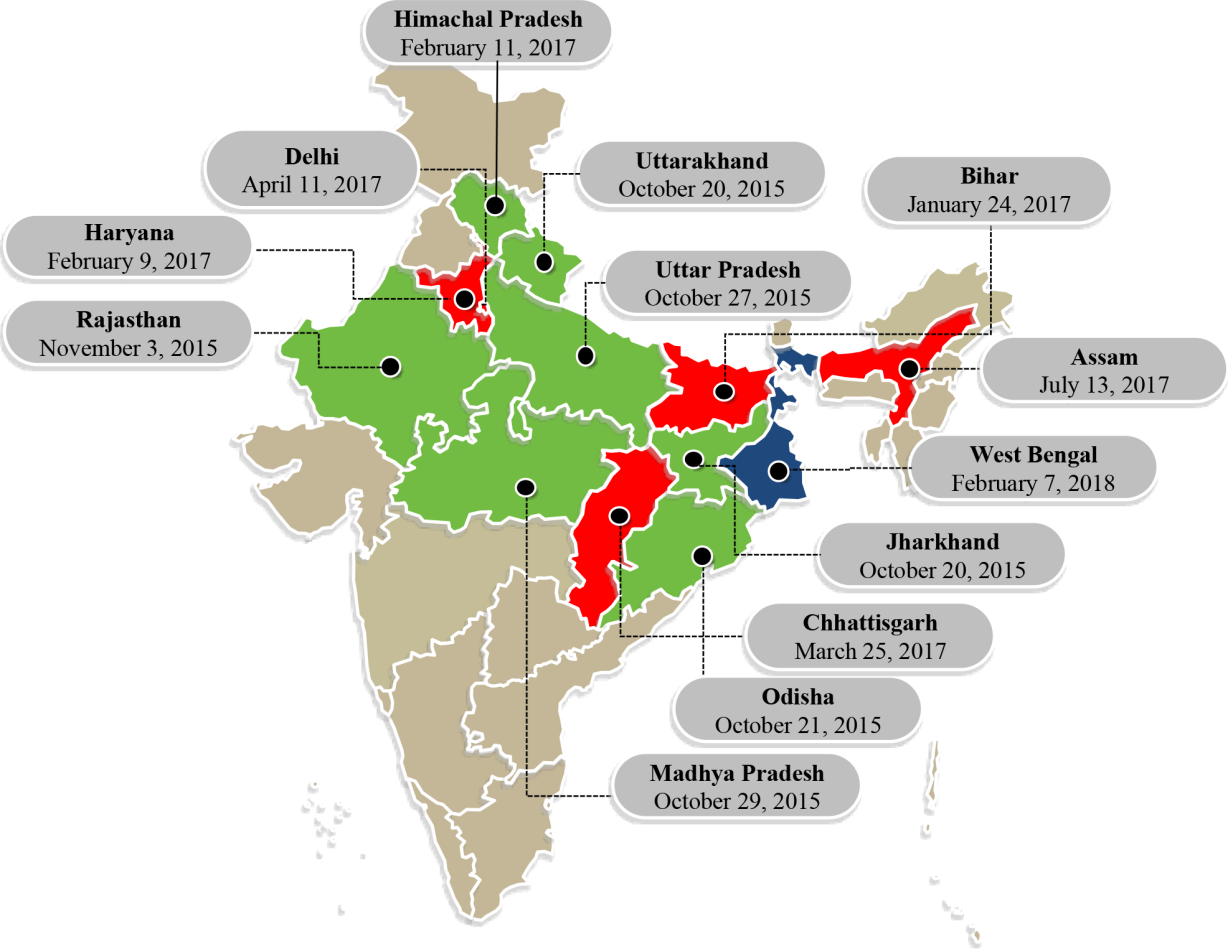
Kilkari program launch dates by State. Colours denote the year of launch for each state: green for 2015, red for 2017 and blue for 2018.

India is home to over 1.3 billion people disbursed across 28 states and eight union territories. The Kilkari programme was designed and piloted by BBC Media Action in the Indian state of Bihar in 2013, and then redesigned and scaled across 13 states in collaboration with the Ministry of Health and Family Welfare (MOHFW) between 2015 and 2019 (figure 1).

### Study population

This study draws on data from two populations of pregnant and postpartum women: (1) Kilkari subscribers in the 13 states where programme implementation was underway as part of the Kilkari national programme supported by BBC Media Action and the MOHFW and (2) women enrolled into an individually RCT in four districts of the central Indian state of Madhya Pradesh. The former are pregnant and postpartum women subscribed to Kilkari based on the mobile number captured in governmental tracking registries called, depending on the state, the Maternal and Child Health Tracking System or Reproductive and Child Health system. Online supplemental table 3 summarises the total population of pregnant women eligible for Kilkari across 13 States. An average of 21% of pregnancies across 13 states was recorded in government tracking registries and, thus, eligible for Kilkari.^18^ Primary data on the demographic profile and health behaviours of Kilkari subscribers are not available across the 13 states where implementation in underway. Accordingly, data on the Kilkari programme’s impact on health outcomes were generalised from an RCT of Kilkari conducted from 2017 to 2020 in the central Indian state of Madhya Pradesh.^19^ This included data on beneficiary reported care-seeking practices and health behaviours. RCT participants (n=5095) were women of 12–34 weeks of gestation at time of enrolment, more than 18years of age, who could speak and understand Hindi, and reported owning or having access to a mobile phone during the day when Kilkari calls were likely to come. These women were identified during a household listing survey described in detail elsewhere.^9 17^

10.1136/bmjgh-2022-009553.supp2 Supplementary data

### Comparators

This study compared women randomised to receive health information messages as part of the Kilkari programme against a status quo of no messages.

Kilkari is free of cost to subscribers. Depending on the timing of enrolment, subscribers may receive up to 72 weekly, stage-based, prerecorded calls about reproductive, maternal, neonatal and child health directly to families’ mobile phones, starting as early as the second trimester of pregnancy and ending when the child is 1-year old.^4^ Across health content areas, 18% of cumulative call content is on family planning (benefits of family planning, modern reversible methods, sterilisation, pregnancy tests); 13% on child immunisations (diseases covered, doses); 13% on nutrition (malnutrition, growth monitoring, maternal anaemia); 12% on infant feeding (quality of food, breastfeeding, complementary feeding, child anaemia); 10% on pregnancy care (antenatal care, institutional delivery, rest, tetanus toxoid, emergency services); 7% on entitlements; 7% on diarrhoea; 7% on postnatal care (newborn danger signs, cord care, hypothermia) and the remainder on a range of topics including intrapartum care, water and sanitation and early childhood development. Additional details on the programme are reported elsewhere.^9 17^

### Perspective and analytic time horizon

Data were collected from a programme perspective for the analytic time horizon of October 2015 to April 2019. The programme perspective includes all costs incurred by the implementing partners in the design and implementation of the Kilkari programme. The programme perspective was selected because it most closely aligns to the costs future payers (Government of India, external donors) would likely incur to introduce and support continued programme implementation. The programme is not anticipated to have resulted in beneficiaries incurring costs to receive calls, nor to the health system since the service is provided directly to the mobile phones of those subscribed drawing from existing government tracking registries. The time horizon used corresponds to the window of time BBC Media Action was supporting the national scale-up of Kilkari. Incremental cost-effectiveness ratios are presented for calendar years 2016–2018 each of which include a full 12 months of programmatic activities.

### Costs

Economic costs were estimated based on financial records maintained by implementing partners including BBC Media Action, the Grameen Foundation, Dimagi and Beehyv. Costs are categorised into capital and recurrent costs and presented for the core ‘ingredients’ or activities which comprise Kilkari. Capital costs included one-time costs associated with infrastructure (third-party hardware and software, hosting telecommunications infrastructure), technology (software licensing, MOTECH engine costs, and IVR professional services fee), content creation and training. Recurrent costs included telecommunication call costs, data centre and technical support, personnel (BBC Media Action, Program Management Unit, management and operations), office space and other miscellaneous costs. Costs were adjusted into 2019 base year US dollars (coinciding with the final year of effect estimates) using local consumer price indices and market exchange rates. Capital costs were annualised over the lifespan of the project using a 3% discount rate.

### Outcomes (selection, measurement, valuation)

Maternal and child (0–12 months) lives saved were the primary the health outcome. Lives saved were derived using the Lives Saved Tool (LiST) which ‘calculates changes in cause-specific mortality based on intervention coverage change, intervention effectiveness for that cause and the percentage of cause-specific mortality sensitive to that intervention’.^20^ Details on the methods underpinning LiST are outlined in detail elsewhere.^20^ The number of lives saved was estimated for each of the 13 states where Kilkari implementation is underway. Estimates of the total population of women eligible for Kilkari were inputted into LiST^18^ along with data on incremental changes in coverage drawn from the Kilkari RCT in Madhya Pradesh.^19^ Coverage estimates were used for only those health behaviours observed to have a statistically significant difference across RCT study arms including modern reversible contraceptive method use, sterilisation and immunisations at 10 weeks. These findings and the underpinning methods used to derive them are presented in detail in a companion paper published elsewhere.^4 6 19^ Online supplemental tables 1–2 presents coverage input estimates used in LiST. Multiple iterations of LiST were run for each state to generate upper and lower bound estimates of lives saved using the 95% CI) around point estimates of coverage for each behaviour.

### Study parameters

Table 1 presents parameters for the 2018 calendar year. Online supplemental tables 3–4 present parameters for 2016 and 2017. High and low estimates for costs are based on a ±10% change around each parameter. For health effects, the upper and lower bounds of the 95% CIs for coverage estimates were used in LiST to generate high and low scenarios.

**Table 1:** Parameters for 2018 Kilkari programme costs and effects for implementation across 13 states

Parameter	Deterministic			Distribution	Probabilistic	Mean	SD
	Base case	High	Low				
Capital costs							
Infrastructure	US$11 103	US$12 213	US$9992	Gamma	11 749	11 103	555
IVR licensing and professional services	US$6 408 731	US$7 049 604	US$5 767 858	Gamma	6 831 600	6 408 731	320 437
Audio content creation	US$2 864 651	US$3 151 116	US$2 578 186	Gamma	2 691 565	2 864 651	143 233
BBC MA Computers	US$11507,88	US$1 265 866	US$1 035 709	Gamma	1 175 398	1 150 788	57 539
Film for training	US$662 571	US$728 828	US$596 314	Gamma	653 843	662 571	33 129
Total capital costs	US$11 097 843	US$122 076	US$99 881	Gamma	10 734 868	11 097 843	554 892
Recurrent costs							
Kilkari call costs	US$59 189 231	US$65 108 155	US$53 270 308	Gamma	56 513 124	59 189 231	2 959 462
BBC MA Personnel	US$65 493 997	US$72 043 397	US$58 944 598	Gamma	68 992 814	65 493 997	3 274 700
Project management unit	US$35 295 492	US$38 825 042	US$31 765 943	Gamma	35 801 942	35 295 492	1 764 775
Technical support	US$23 929 707	US$26 322 678	US$21 536 737	Gamma	22 593 596	23 929 707	1 196 485
Indirect costs	US$7 024 939	US$7 727 433	US$6 322 445	Gamma	6 507 149	7 024 939	351 247
BBC MA office costs	US$5 422 154	US$5 964 370	US$4 879 939	Gamma	6 010 461	5 422 154	271 108
BBC MA management fees	US$4 736 346	US$5 209 980	US$4 262 711	Gamma	4 853 924	4 736 346	236 817
Travel	US$3 373 255	US$3 710 580	US$3 035 929	Gamma	3 470 423	3 373 255	168 663
Other costs: donor audit, taxes, misc.	US$4 522 038	US$4 974 242	US$4 069 835	Gamma	4 396 843	4 522 038	226 102
Communications	US$322 905	US$355 195	US$290 614	Gamma	315 278	322 905	16 145
Dissemination, workshops	US$1 732 441	US$1 905 685	US$1 559 197	Gamma	1 883 666	1 732 441	86 622
Total recurrent costs	US$211 042 506	US$2 321 468	US$1 899 383	Gamma	218 072 310	211 042 506	10 552 125
Total costs	US$222 140 349	US$244 354 384	US$199 926 314	Gamma	220 679 456	222 140 349	11 107 017
Maternal lives saved				Distribution			
Assam	39	52	45	Lognormal	38,30	39,00	1,75
Bihar	14	15	13	Lognormal	13,21	14,00	0,50
Chattisgarh	16	17	14	Lognormal	16,05	16,00	0,75
Delhi	25	27	22	Lognormal	25,06	25,00	1,25
Haryana	18	19	16	Lognormal	16,99	18,00	0,75
Himachal Pradesh	10	28	9	Lognormal	12,03	10,00	4,75
Jharkhand	2	2	2	Lognormal	2,00	2,00	-
Madhya Pradesh	30	32	27	Lognormal	30,87	30,00	1,25
Odisha	25	26	22	Lognormal	26,13	25,00	1,00
Rajasthan	39	41	35	Lognormal	39,60	39,00	1,50
Uttarakhand	8	8	7	Lognormal	8,53	8,00	0,25
Uttar Pradesh	48	51	43	Lognormal	44,19	48,00	2,00
Total maternal lives saved	274	318	255	Lognormal	295,66	274,00	15,75
<5 lives saved							
Assam	623	821	704	Lognormal	592,66	623,00	29,25
Bihar	213	229	189	Lognormal	221,59	213,00	10,00
Chattisgarh	245	263	217	Lognormal	255,02	245,00	11,50
Delhi	394	424	350	Lognormal	375,13	394,00	18,50
Haryana	266	286	236	Lognormal	266,07	266,00	12,50
Himachal Pradesh	148	159	131	Lognormal	145,52	148,00	7,00
Jharkhand	41	44	37	Lognormal	39,42	41,00	1,75
Madhya Pradesh	684	724	607	Lognormal	681,50	684,00	29,25
Odisha	590	623	526	Lognormal	589,36	590,00	24,25
Rajasthan	885	937	785	Lognormal	963,95	885,00	38,00
Uttarakhand	179	189	159	Lognormal	172,35	179,00	7,50
Uttar Pradesh	1128	1196	999	Lognormal	1 139,76	1 128,00	49,25
Total <5 lives saved	5396	5895	4940	Lognormal	5 372,68	5 396,00	238,75
Total lives saved	5670	6213	5195	Lognormal	5 634,80	5 670,00	254,50

### Analytics

Analyses used to estimate differences in coverage for target health behaviours are described in depth elsewhere.^19^ In brief, to assess exposure to Kilkari content, call data records from the IVR system were linked to baseline and endline survey data. Listening patterns were assessed for each subscriber by call, for the duration of their subscription to Kilkari using call data records. The latter provides evidence on subscriber engagement with calls, including the duration of listening to individual calls. To link individual call listening patterns to health outcomes, we mapped the content of calls to key outcome indicators measured in household surveys. Exposure was defined at a listening threshold of 50% or more of the cumulative duration of the calls mapped to the outcome. Primary analyses of outcomes were done with modified intention-to-treat (ITT) analyses at the individual level, so that outcomes were analysed regardless of the degree of listening to Kilkari. To assess the impact of exposure on outcomes, compliance-adjusted treatment effects (CATE) were additionally generated using the instrumental variable methodology.^4 6^ Coverage estimates were inputted into LiST and scenarios run for each state which considered the duration of implementation, total fertility rate and Kilkari-adjusted population coverage. Base case estimates for lives saved are based on ITT estimates of coverage; arguably the most conservative approach.

To characterise heterogeneity, we additionally estimated the number of lives saved across socioeconomic strata, and similarly, based on Kilkari exposure, drawing from CATE findings. Socioeconomic strata were derived using principal components analyses. Cost data by subgroup were not available and, therefore, estimates of cost-effectiveness by subgroup not generated.

Statistical analyses were carried out in R^21^ and Microsoft excel (Microsoft, Redmond, Washington). The impact of uncertainty was assessed through probabilistic sensitivity analysis, using standard Monte-Carlo simulation resampling.^22^ In this method, data points were randomly sampled from original data, with replacement, and ICERs were calculated. This process was repeated to represent what results might arise if a large number of similar trials were performed. These calculations were performed in Excel using a Visual Basic-based macro to perform the resample automatically. In total, 1000 iterations were generated for each simulation and plotted on two-dimensional cost-effectiveness planes. Results were presented as cost-effectiveness acceptability curves, which are standardised tools for summarising the probability of cost-effectiveness based on variations in the ceiling ratio.^23^ We additionally evaluated findings against a ceiling ratio based on the per capita gross domestic product (GDP) for India in 2018 (US$1998)^24^; a threshold favoured by Commission on Macroeconomics and Health for assessing cost-utility analyses using disability-adjusted life years (DALYs) as the primary outcome. In the absence of a similar standard for cost-effectiveness analyses^25^ which use lives saved as the primary outcome, we present this threshold as a conservative proxy in addition to the cost-effectiveness acceptability curves, which allow users to weigh results against a range of alternative willingness to pay thresholds.

### Role of the funding source

The Bill and Melinda Gates Foundation had no role in the study design; collection, analysis and interpretation of data; in the writing of the report or in the decision to submit the paper for publication. All authors confirm that they had full access to all the data in the study and accept responsibility for the publication submitted.

### Patient and public involvement

This is a secondary analysis which draws on primary data collected as part of the Kilkari RCT in Madhya Pradesh. As part of the RCT, beneficiaries were engaged during household surveys and qualitative interviews. The latter included engaging a small number of patients in the refinement of survey tools. Unfortunately because of COVID-19 and associated travel restrictions, patients could not be involved in the dissemination of study findings. However, public dissemination of the results has occurred through a number of presentations in India and elsewhere globally.

## Results

### Summary of main results

Table 2 summarises programme costs for the 2015–2019 window. Overall, capital costs were an average of 12% of total programme costs, while recurrent costs comprised 88%. Kilkari call costs constituted 23% of total costs, followed by costs associated with BBC Media Action personnel (22%), the programme management unit (15%) and other technical support (12%). Among capital costs, infrastructure (6% of total costs) and IVR licensing and professional services (2%) were the leading cost drivers.

**Table 2:** Total programme costs (USD) incurred to support implementation and expansion to 13 states from 2015 to 2019

	2015	2016	2017	2018	2019	2015–2019	% of total
Months of implementation by year	4	12	12	12	4	44	
Capital costs							
Infrastructure	$170 379	$170 417	$170 454	$111	$111	$511 472	6
IVR licensing and professional services	$ -	$64 028	$64 087	$64 087	$59	$192 262	2
Audio content creation	$6 626	$14 543	$34 667	$28 647	$20 730	$105 212	1
Data centre	$28 949	$28 949	$28 949	$ -	$ -	$86 847	1
Technology support	$26 061	$26 061	$26 061	$ -	$ -	$ 78 184	1
BBC MA computers	$75	$2 266	$3 785	$11 508	$9318	$26 951	0
Film for training	$ -	$ -	$ -	$6626	$6626	$13 251	0
Total capital costs	$232 092	$306 264	$328 003	$110 978	$36 843	$1 014 180	12
Recurrent costs							
Kilkari call costs	$100 801	$633 701	$639 658	$591 892	$ -	$1 966 052	23
BBC MA personnel	$153 373	$386 194	$545 363	$654 940	$136 666	$1 876 536	22
Project management unit	$47 296	$412 347	$464 645	$352 955	$ -	$1 277 243	15
Technical support	$81 886	$265 227	$282 735	$239 297	$118 625	$987 771	12
Indirect costs	$50 186	$73 955	$101 056	$70 249		$295 447	4
BBC MA office costs	$70 224	$112 736	$98 178	$54 222	$6707	$342 066	4
BBC MA management fees	$89 716	$69 572	$39 910	$47 363	$36 825	$283 386	3
Travel	$27 552	$39 862	$44 356	$33 733	$3717	$149 220	2
Other costs: donor audit, taxes, miscellaneous	$9490	$36 736	$60 644	$45 220	$ -	$152 090	2
Communications	$5290	$4972	$6857	$3229	$ -	$20 348	0
BBC MA legal fees	$ -	$ -	$ -	$ -	$4 971	$4 971	0
Dissemination, workshops	$ -	$ -	$3 137	$17 324	$10 061	$30 522	0
Total recurrent costs	$635 813	$2 035 302	$2 286 539	$2 110 425	$317 573	$7 385 652	88
Total costs	$867 905	$2 341 566	$2 614 542	$2 221 403	$354 416	$8 399 832	100

IVR, interactive voice response.

Table 3 summarises total lives saved by state and year, adjusted for duration of implementation. Six states (Jharkhand, Madhya Pradesh, Odisha, Rajasthan, Uttarakhand and Uttar Pradesh) had over 1200 days of implementation and comprised the largest proportion of lives saved. One-fifth (25%) of total lives saved came from Uttar Pradesh, followed by Rajasthan (20%) and Madhya Pradesh (15%). The Indian state of West Bengal launched in February of 2018 and as a result, had the lowest (4%) overall proportion of lives saved. The majority of lives saved (96%) occurred in children 0–12 months with the remaining (4%) attributed to maternal lives saved.

**Table 3:** LiST estimates of the number of lives saved by State 2015–2019

State	Estimated days of implementation	2015	2016	2017	2018	2019	Total lives saved	% of total lives saved
Assam	627	0	0	0	662	198	860	6
Bihar	797	0	0	0	227	68	295	2
Chattisgarh	768	0	0	0	261	76	337	2
Delhi	720	0	0	0	419	124	543	4
Haryana	766	0	0	0	284	84	368	3
Himachal Pradesh	764	0	0	0	158	46	204	1
Jharkhand	1256	0	30	39	43	12	124	1
Madhya Pradesh	1247	0	540	661	714	187	2102	15
Odisha	1255	0	402	547	615	163	1727	12
Rajasthan	1243	0	700	853	924	242	2719	20
Uttarakhand	1243	0	146	175	187	49	557	4
Uttar Pradesh	1251	0	874	1083	1176	308	3441	25
West Bengal	417				0	566	566	4
Total	4866	–	2692	3358	5670	2122	13 842	100

Table 4 summarises incremental cost-effectiveness ratios for lives saved for 2016–2018. Results suggest that the cost-effectiveness of Kilkari improved with time and scale. By 2018, when implementation was underway in 13 states, the average incremental cost per live saved was US$391.78 ($384.84–$393.30) as compared with US$953.29 ($889.21–$1091.56) in 2016 when implementation was only newly underway in seven states.

**Table 4:** Incremental cost-effectiveness ratios 2016–2018

	Incremental costs ($USD)	Incremental lives saved	Cost per live saved
			(US$)
2016			
Kilkari vs Status quo	$2 566 259	2692	$953.29
			($889.21–1091.56)
2017			
Kilkari vs Status quo	$2 871 906	3358	$855.24
			($805.36–967.62)
2018			
Kilkari vs Status quo	$2 221 403	5670	$391.78
			($384.84–$393.30)

### Effect of uncertainty

**Figure 2:**
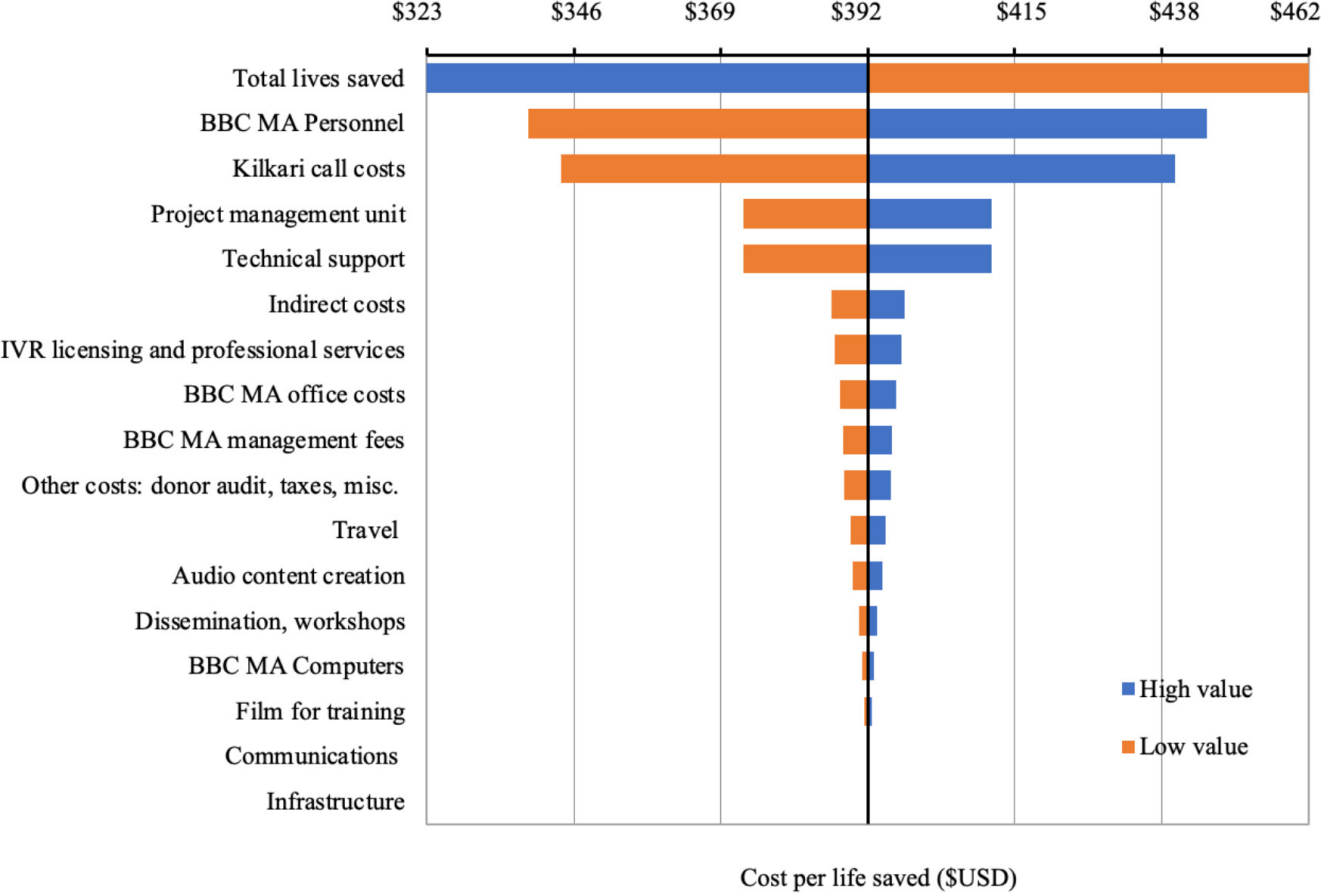
Tornado diagram of 2018 costs and lives saved.

**Figure 3:**
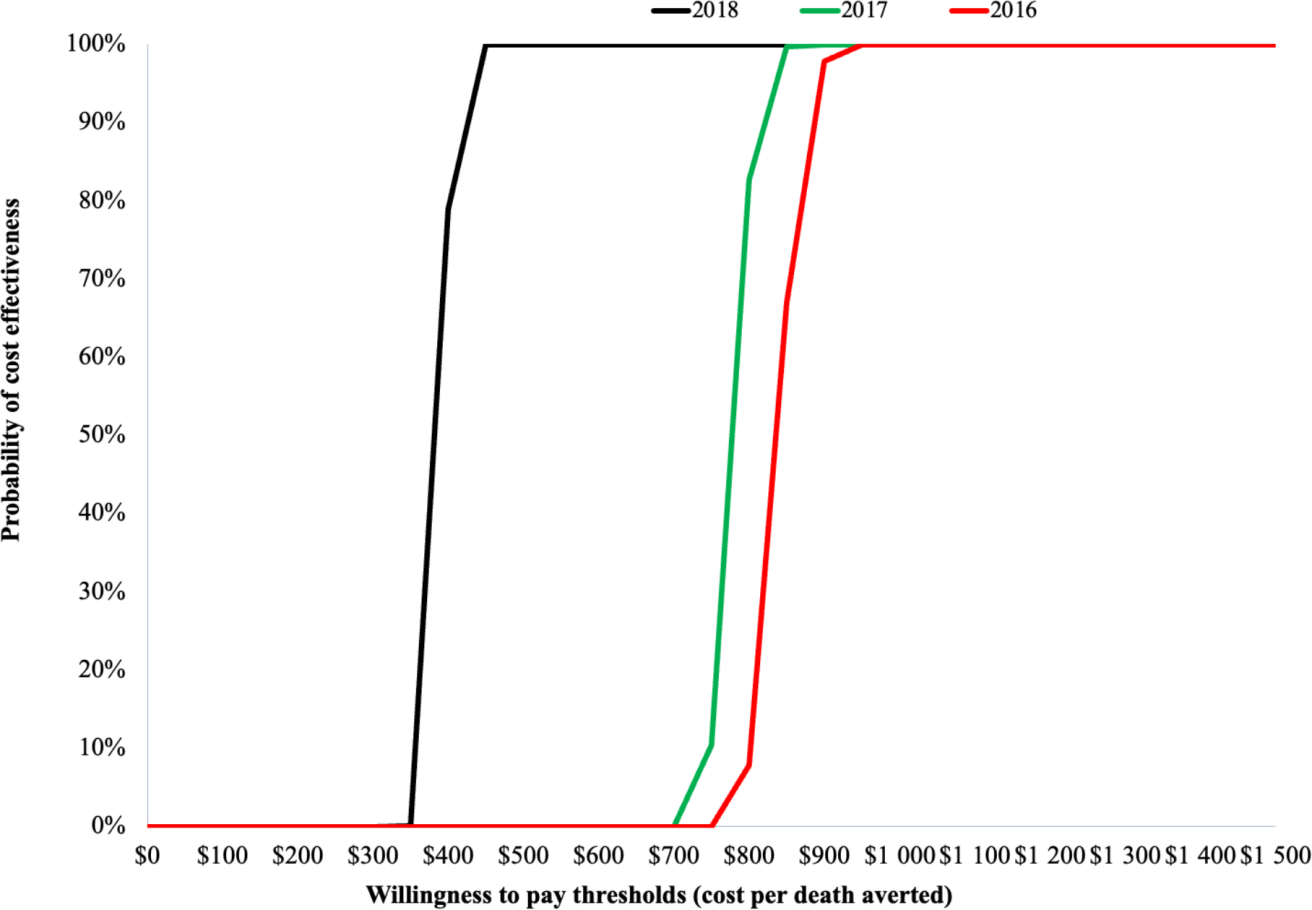
Willingness to pay (WTP) to avert maternal and child mortality.

Figure 2 presents a tornado diagram for 2018 costs and lives saved. The leading drivers of cost-effectiveness were technology and call costs, followed by programme personnel costs to manage and support the programme. Online supplemental figure 1 depicts the cost-effectiveness plane, while figure 3 presents cost-effectiveness acceptability curves for 2016–2018. With a cost per live saved of US$391.78 in 2018, Kilkari falls beneath the World Bank’s GDP per capita threshold of US$1998 for 2018.

10.1136/bmjgh-2022-009553.supp1 Supplementary data

## Discussion

Study findings present evidence on the value for money of Kilkari—one of the world’s largest direct to beneficiary mobile health service. This study is the first of its kind conducted of a digital health programme being implemented at scale in India and elsewhere globally. Findings suggest that an estimated 13 842 lives were saved across 13 states from October 2015 to April 2019; 25% of these are estimated to have occurred in the Indian state of Uttar Pradesh. From October 2015 to April 2019, nearly US$8.4 million was spent to support the introduction and ongoing implementation of Kilkari across 13 states. IVR call costs were the leading driver of costs (23%). The incremental cost per life saved ranged from US$953 in 2016 to US$392 in 2018. The incremental cost-effectiveness ratio decreased over time and with increasing scale. Based on these findings, Kilkari is highly cost-effective using a GDP threshold and compares favourably with other low-cost high priority interventions.^26^

Estimates on the number of incremental lives saved were derived using LiST. LiST is a mathematical modelling tool which allows users to estimate the impact of changes in coverage for reproductive maternal newborn and child health interventions on mortality in low and middle-income countries.^20^ LiST has been featured in over 150 peer review publications^27^ and used to model the impact interventions which may have on mortality in a range of settings globally including Afghanistan, Bangladesh,^28^ Malawi,^29^ Mozambique, Niger^30^ and South Africa. In the context digital health solutions, it has been used in economic evaluations to measure the number of lives saved as result of a frontline health worker application in Bangladesh^28^ and direct to beneficiary solutions in Ghana^16^ and South Africa.^14^ The external validity of LiST has been assessed through comparisons with alternative data sources including measured data from vector control studies,^31^ demographic and health survey data^32^ and community-based intervention trials.^33^ The external validity of LiST with regards to the estimated impact of changes in coverage of reproductive health interventions has not yet been assessed to our knowledge. While this absence of more comprehensive data on LiST’s external validity is a limitation, others have sought to highlight the importance such tools may nevertheless have particularly in modelling effects that RCTs cannot reasonably be expected to detect.^34^ In the context of interventions like Kilkari, the impact on mortality is likely to fall under 5%. Detecting such a small margin of change would be cost prohibitive and simply infeasible given larger global funding challenges for digital health programmes and their evaluations.

Table 5 presents a league table contextualising findings with estimates drawn from the literature. In the absence of comparable data using lives saved as an outcome, we have relied on comparisons with publications which present data on the cost per DALYs averted. DALYs are a summary measure of overall disease burden expressed in terms of years lost due to ill-health, disability or early death.^35^ In the absence of disability weights for the full range of health conditions considered in our analyses,^36^ we have not presented DALYs as an outcome measure. Nevertheless, comparisons with our findings may provide some insights into how Kilkari compares with alternative resource uses.

**Table 5:** League table

Comparison with other interventions in the literature	Description	Cost per DALY averted (USD)	Source
Treat severe malaria with artesunate vs quinine, Africa and Southeast Asia	Use of parenteral artesunate to treat children with severe malaria in Africa and Southeast Asia	$5	26
Kilkari	Maternal mobile health information messages	$26–$36	
Zinc added to oral rehydration therapy	Used zinc as adjunct therapy to standard treatment of acute childhood diarrhoea	$10–50	26
Community management severe-acute malnutrition	Community-based therapeutic care: Diagnosis, RUTF (Ready-to-Use-Therapeutic Food), supplements, in-patient treatments, out-patient visits, weekly follow-ups	$25–40	26
Rotavirus immunisation in India	The public health impact, cost, and cost-effectiveness of universal vaccination in India using the 116E vaccine	$56	39
	Extended cost-effectiveness analysis' of a hypothetical publicly financed programme for rotavirus vaccination in India	$66	40
Innovative Mobile Technology for Community Health Operation (ImTeCHO) in Gujarat, India	Job aid for Accredited Social Health Activists (ASHAs) and staff of primary health centres to increase coverage of maternal, neonatal, and child healthcare.	$74 per life-years saved $5057 per death averted	41
Maternal and neonatal care at home	Maternal and neonatal services delivered at home, with community mobilisation and health system strengthening	$13–126	26
Original EPI-6 plus Hepatitis B	Expanded Program of Immunisation with six vaccines	$103	26
Pneumococcus and rotavirus low income countries	Implementing pneumococcus and rotavirus vaccination programme; low-income countries are eligible to procure vaccines from Gavi at low prices	$103	26
Handwashing BCC (behaviour change communications)	Increase hand-washing after handling child stool and disposal of stool in latrines	$90–225	26
MAMA South Africa	Maternal mobile health information messages	$200-$1985 per DALY averted $5652 - $56 011 per live saved	14
Haemophilus influenza *type b* (HiB) vaccine—India, Gujarat	Nationwide Hib vaccination in India	$155-US$939	42

Comparisons of results observed with other digital health programmes are challenging given the limited number of studies present in the literature, variations in the programmes being evaluated and heterogeneity in the methodological approaches undertaken. Among the other large-scale direct to beneficiary mobile health solutions, evidence on value for money was available only for the MAMA project in South Africa.^14^ MAMA was one of the precursor programmes to MomConnect^5 15^—the National Department of Health’s flagship mobile messaging programme which sent up to 140 text messages to new and expectant women attending pregnancy services in the public sector.^15^ Efforts to determine the cost-effectiveness of MAMA were conducted as part of a retrospective study in six health centres in Gauteng province and sought to gradually model the implications in terms of costs and effects of scaling-up services to pregnant women throughout Gauteng province, South Africa from 2012 to 2017.^14^ Results suggested that the incremental costs per live from a societal perspective ranged from US$56 011 in year 1 of implementation to US$5652 in the fifth year.^14^ Findings from the MAMA study compare to those observed in our study (cost per live saved of US $392-$953) and suggest that with increasing population-level coverage and expansion, the programme’s cost-effectiveness improves. Similar to findings in our study, the leading drivers of cost for MAMA were call costs (31%).^14^

Despite the comparability of findings, some methodological differences in the approach undertaken to assessing cost-effectiveness are noteworthy. First, economic costs in the MAMA study were estimated from a societal perspective inclusive of programme, health systems and user costs for a 5-year analytic time horizon (2012–2017). Collecting data from a societal perspective was not possible for Kilkari and instead a programme perspective was taken. In contrast to MAMA which was provided through government health facilities and depended on government providers to register subscribers, Kilkari was provided directly to beneficiaries and did not rely on added health system inputs including provider time to support registration. Instead, the subscriber population for Kilkari was drawn from secondary data within government tracking registries including the last menstrual period for women and their phone number. This programmatic strategy of enrolment based on government tracking registry data was designed to facilitate programme expansion and scalability, while limiting the burden placed on public sector providers. In terms of beneficiary costs, direct to beneficiary mobile health programmes may have cost implications for subscribers on two fronts: (1) bolstering utilisation of health services and in turn, costs for transport, wages lost, child care, etc and (2) costs to receive health information content, including hardware and phone credit. Both programmes were provided free of cost to beneficiaries. The MAMA study sought to attribute a monetary value to changes in health service utilisation, which was appropriate given that the health outcomes assessed were linked to pregnancy and immunisation care seeking in the public sector. In the context of Kilkari, limitations in the total interview time available to administer structured surveys to women enrolled into the RCT meant that questions on direct and indirect costs to beneficiaries for health careseeking were not possible to capture. Beyond the measurement of costs, health effects in both studies were derived via a similar approach of inputting coverage data into LiST and modelling lives saved. However, the quality of evidence differed markedly across studies. Kilkari data were drawn from a large (n=5095) individual RCT implemented across four districts in Madhya Pradesh, whereas MAMA impact estimates were drawn from a small-scale (<200 mother–infant pairs) retrospective case-control study conducted in six health clinics in Gauteng province.^37^ The MAMA study further sought to forecast the potential costs and consequences of programme expansion based on this limited primary data. In the context of Kilkari, primary data on costs of programme expansion across 13 states were used.

### Limitations

Estimates of health effects were modelled using LiST and based on incremental changes in coverage observed as part of an RCT conducted in four districts of the central Indian state of Madhya Pradesh. While RCT findings provide the most definitive evidence to date on the impact of Kilkari, they nevertheless may not be representative of the programme’s impact in other contexts. India is extremely diverse and states such as Bihar and Uttar Pradesh tend to perform below Madhya Pradesh for most maternal and child health care seeking and health behaviours. State-level variations in mobile phone access and use, particularly among women, may too have implications for programme reach, exposure and impact.^38^ We further note that data on Kilkari’s impact on health outcomes related to family planning are based on self-reported use contraceptive methods, which may be subjected to social desirability bias. There additionally could be information and recall biases since these data were collected at a single timepoint (12 month’s postpartum) and yet pertain to practices occurring during the 12-month window preceding the interview. Cost estimates are presented from a programme perspective. Costs to beneficiaries include the cost of owning the handset and data, along with potential costs incurred in care seeking for target health behaviours. These costs were not possible to collect as part of the RCT in Madhya Pradesh due to constraints in the number of questions possible to ask within a limited interview window. Additional costs to the health system were similarly not captured but are likely minimal. These are likely to include frontline health worker time costs to collect and register the phone numbers for couples as well as costs associated with increases in care seeking as a result of exposure to health information messages, which might bolster beneficiary awareness. Frontline health workers register couples as part of routine health information systems data collected—programme activities have not led to a modification in this pre-existing behaviour, rather they simply leverage existing data as the sampling frame.

## Conclusion

From 2016–2018, the 13-state implementation of the Kilkari programme was associated with a cost per live saved of US$392–US$953. These findings suggest that Kilkari is highly cost-effective and compares favourably with alternative resource uses for maternal and child health in India. This study contributes to the limited evidence base on the value for money of digital health solutions.

10.1136/bmjgh-2022-009553.supp3 Supplementary data

## Data Availability

Data are available upon reasonable request. Data are available upon reasonable request to the study PI Dr. Amnesty LeFevre aelefevre@gmail.com.
