# Supplementary material for: Cost-effectiveness of a direct to beneficiary mobile communication programme in improving reproductive and child health outcomes in India
Source: BMJ Glob Health. 2023 Mar 23;6(Suppl 5):e009553. doi: 10.1136/bmjgh-2022-009553 (PMC10175950; doi:10.1136/bmjgh-2022-009553)
Supplement: Supplementary data [file bmjgh-2022-009553supp002.pdf]

Supplementary Table 1. Coverage estimates inputted into LiST drawn from the Kilkari Impact Evaluation in four districts of Madhya Pradesh

Health behaviours	Coverage across study arms						Coverage across exposed vs. not exposed						Incremental change in coverage					
	Intervention			Comparison			Exposed			Not exposed			Intervention - Comparison			Exposed - Not exposed		
	Base case	UL	LL	Base case	UL	LL	Base case	UL	LL	Base case	UL	LL	Base case	UL	LL	Base case	UL	LL
Child 10 week immunisations	75%	73%	77%	72%	70%	74%	75%	72%	78%	73%	72%	74%	3%	3%	3%	2%	1%	4%
Condom use	27%	24%	29%	24%	22%	26%	34%	30%	38%	24%	23%	26%	3%	2%	3%	10%	7%	13%
Female sterilisation	13%	12%	14%	15%	13%	16%	10%	8%	13%	14%	13%	15%	-2%	-2%	-2%	-4%	-5%	-2%
Male sterilisation	0%	0%	1%	0%	0%	0%	0%	0%	1%	0%	0%	0%	0%	0%	0%	0%	0%	0%
Injectable	2%	1%	2%	2%	1%	2%	3%	2%	5%	1%	1%	2%	0%	0%	0%	2%	1%	4%
Intrauterine device	3%	3%	4%	3%	2%	4%	4%	3%	6%	3%	3%	4%	0%	0%	0%	1%	0%	3%
Oral contraceptive pill	5%	4%	6%	4%	3%	5%	5%	3%	7%	4%	4%	5%	1%	1%	1%	0%	0%	2%
Withdrawal	17%	16%	19%	17%	15%	19%	20%	16%	23%	17%	16%	18%	0%	0%	0%	3%	1%	6%
Abstinence	22%	20%	23%	22%	20%	23%	22%	19%	26%	22%	20%	23%	0%	0%	0%	0%	-2%	3%
Rhythm method	30%	28%	32%	30%	28%	32%	30%	27%	35%	30%	29%	31%	0%	0%	0%	0%	-2%	3%

UL: Upper limit
LL: Lower limit

Supplementary Table 2. LiST population input parameters

State	Total population	Eligible* for Kilkari	Eligibility adjusted population
Assam	55 218 267	46%	25 400 403
Bihar	175 836 168	3%	5 275 085
Chattisgarh	55 677 340	19%	10 578 695
Delhi	29 262 861	77%	22 532 403
Haryana	43 523 846	30%	13 057 154
Himachal Pradesh	11 784 520	66%	7 777 783
Jharkhand	49 841 153	2%	996 823
Madhya Pradesh	109 730 483	18%	19 751 487
Odisha	63 418 058	32%	20 293 779
Rajasthan	104 460 771	25%	26 115 193
Uttarakhand	15 370 472	36%	5 533 370
Uttar Pradesh	301 892 716	8%	24 151 417
West Bengal	156 817 423	75%	117 613 067
**Total**	**1 172 834 078**		**299 076 658**

* Eligibility is defined as the percentage of pregnancies included in government tracking registries

Supplementary Table 3. Parameters for 2016 Kilkari program costs and effects for implementation across 13 states

Parameter	Deterministic Base case	High	Low	Distribution	Probabilitistic	Mean	SD
**Capital costs**							
Infrastructure	$ 170 417	$ 187 458,15	$ 153 374,85	Gamma	155 122,88	170 416,50	8 520,83
IVR licensing and professional services	$ 64 028	$ 70 430,69	$ 57 625,11	Gamma	61 563,29	64 027,90	3 201,40
Audio content creation	$ 14 543	$ 15 997,53	$ 13 088,89	Gamma	14 143,01	14 543,21	727,16
Data center	$ 28 949	$ 31 844,00	$ 26 054,18	Gamma	28 899,36	28 949,09	1 447,45
Technology support	$ 26 061	$ 28 667,60	$ 23 455,31	Gamma	25 960,97	26 061,45	1 303,07
BBC MA Computers	$ 2 266	$ 2 492,09	$ 2 038,98	Gamma	2 089,66	2 265,53	113,28
**Total capital costs**	**$ 306 264**	**$ 336 890**	**$ 275 637**	Gamma	308 969,46	306 263,69	15 313,18
**Recurrent costs**							
Kilkari call costs	$ 633 701	$ 697 071	$ 570 331	Gamma	613 693,95	633 701,01	31 685,05
BBC MA Personnel	$ 386 194	$ 424 814	$ 347 575	Gamma	353 545,36	386 194,12	19 309,71
Project management unit	$ 412 347	$ 453 581	$ 371 112	Gamma	371 970,55	412 346,73	20 617,34
Technical support	$ 265 227	$ 291 750	$ 238 705	Gamma	252 802,15	265 227,33	13 261,37
Indirect costs	$ 73 955	$ 81 351	$ 66 560	Gamma	74 408,22	73 955,26	3 697,76
BBC MA office costs	$ 112 736	$ 124 009	$ 101 462	Gamma	110 065,58	112 735,50	5 636,78
BBC MA management fees	$ 69 572	$ 76 529	$ 62 615	Gamma	72 144,58	69 571,85	3 478,59
Travel	$ 39 862	$ 43 849	$ 35 876	Gamma	40 959,62	39 862,49	1 993,12
Other costs: donor audit, taxes, misc.	$ 36 736	$ 40 410	$ 33 063	Gamma	35 625,29	36 736,23	1 836,81
Communications	$ 4 972	$ 5 469	$ 4 475	Gamma	4 593,45	4 971,82	248,59
**Total recurrent costs**	**$ 2 035 302**	**$ 2 238 833**	**$ 1 831 772**	**Gamma**	**2 198 571,31**	**2 035 302,34**	**101 765,12**
**Total costs**	**$ 2 566 259**	**$ 2 822 885**	**$ 2 309 633**	**Gamma**	**2 594 245,39**	**2 566 259,18**	**128 312,96**
**Maternal lives saved**				**Distribution**			
Jharkhand	2	2	1	Lognormal	2,20	2	0,25
Madhya Pradesh	30	32	26	Lognormal	31,66	30	1,50
Odisha	22	23	19	Lognormal	22,59	22	1,00
Rajasthan	39	42	34	Lognormal	38,96	39	2,00
Uttarakhand	8	9	7	Lognormal	8,89	8	0,50
Uttar Pradesh	48	51	42	Lognormal	49,41	48	2,25
Total maternal lives saved	**149,00**	**159,00**	**129,00**	Lognormal	167,75	149	7,50
**<5 lives saved**							
Jharkhand	28	30	25	Lognormal	29,60	28	1,25
Madhya Pradesh	510	547	444	Lognormal	504,63	510	25,75
Odisha	380	405	338	Lognormal	384,68	380	16,75
Rajasthan	661	709	575	Lognormal	716,86	661	33,50
Uttarakhand	138	148	120	Lognormal	130,81	138	7,00

Uttar Pradesh	826	888	720	Lognormal	912,07	826	42,00
Total <5 lives saved	2 543,00	2 727,00	2 222,00	Lognormal	2 809,90	2543	126,25
**Total lives saved**	**2 692,00**	**2 886,00**	**2 351,00**	**Lognormal**	**2 937,32**	**2692**	**133,75**

Supplementary Table 4. Parameters for 2017 Kilkari program costs and effects for implementation across 13 states

Parameter	Deterministic Base case	High	Low	Distribution	Probabilitistic	Mean	SD
**Capital costs**							
Infrastructure	$170 454	$187 498,86	$153 408,16	Gamma	$154 306,62	$170 453,51	$8 522,68
IVR licensing and professional services	$64 087	$70 496,04	$57 678,58	Gamma	$65 823,20	$64 087,31	$3 204,37
Audio content creation	$34 667	$38 133,25	$31 199,93	Gamma	$36 667,86	$34 666,59	$1 733,33
Data center	$28 949	$31 844,00	$26 054,18	Gamma	$28 696,04	$28 949,09	$1 447,45
Technology support	$26 061	$28 667,60	$23 455,31	Gamma	$26 876,36	$26 061,45	$1 303,07
BBC MA Computers	$3 785	$4 163,14	$3 406,21	Gamma	$3 471,88	$3 784,67	$189,23
**Total capital costs**	$328 003	$327 972	$268 341	Gamma	332 617,84	328 002,62	14 907,82
**Recurrent costs**							
Kilkari call costs	$639 658	$703 624,03	$575 692,39	Gamma	$742 440,64	$639 658,21	$31 982,91
BBC MA Personnel	$545 363	$599 899,17	$490 826,59	Gamma	$543 134,31	$545 362,88	$27 268,14
Project management unit	$464 645	$511 109,28	$418 180,32	Gamma	$459 413,13	$464 644,80	$23 232,24
Technical support	$282 735	$311 008,78	$254 461,73	Gamma	$267 605,41	$282 735,25	$14 136,76
Indirect costs	$101 056	$111 161,63	$90 950,43	Gamma	$96 530,35	$101 056,03	$5 052,80
BBC MA office costs	$98 178	$107 995,60	$88 360,04	Gamma	$90 693,99	$98 177,82	$4 908,89
BBC MA management fees	$39 910	$43 901,30	$35 919,24	Gamma	$41 205,29	$39 910,27	$1 995,51
Travel	$44 356	$48 791,54	$39 920,35	Gamma	$42 039,13	$44 355,95	$2 217,80
Other costs: donor audit, taxes, misc.	$60 644	$66 708,28	$54 579,50	Gamma	$62 446,05	$60 643,89	$3 032,19
Communications	$6 857	$7 542,82	$6 171,40	Gamma	$6 658,09	$6 857,11	$342,86
Dissemination, workshops	$3 137	$3 450,53	$2 823,16	Gamma	$3 105,92	$3 136,84	$156,84
**Total recurrent costs**	$2 286 539	$2 437 491	$1 994 311	Gamma	2 332 531,82	2 286 539,04	110 795,06
**Total costs**	$2 871 906,45	$3 126 266,35	$2 557 854,29	Gamma	$2 994 790,84	$2 871 906,45	$142 103,02
**Maternal lives saved**				Distribution			
Jharkhand	1	2	1	Lognormal	0,84	1	0,25
Madhya Pradesh	30	31	26	Lognormal	29,53	30	1,25
Odisha	24	25	21	Lognormal	23,92	24	1,00
Rajasthan	38	40	34	Lognormal	37,23	38	1,50
Uttarakhand	8	9	7	Lognormal	7,43	8	0,50
Uttar Pradesh	47	50	42	Lognormal	46,12	47	2,00
Total maternal lives saved	148	157	131	Lognormal	145,64	148	6,50
**<5 lives saved**							
Jharkhand	38	40	34	Lognormal	39,41	38	1,50
Madhya Pradesh	631	670	557	Lognormal	608,22	631	28,25
Odisha	523	554	466	Lognormal	547,36	523	22,00

Rajasthan	815	866	719	Lognormal	777,20	815	36,75
Uttarakhand	167	177	148	Lognormal	172,21	167	7,25
Uttar Pradesh	1 036	1 102	913	Lognormal	999,04	1036	47,25
Total <5 lives saved	3 210	3 409	2 837	Lognormal	3 390,21	3210	143,00
**Total lives saved**	3 358	3 566	2 968	Lognormal	3 299,36	3358	149,50
