# Supplementary figures and images for: Cost-effectiveness of a direct to beneficiary mobile communication programme in improving reproductive and child health outcomes in India
Source: BMJ Glob Health. 2023 Mar 23;6(Suppl 5):e009553. doi: 10.1136/bmjgh-2022-009553 (PMC10175950; doi:10.1136/bmjgh-2022-009553)

Supplementary Figure 1. Cost effectiveness plane for 2018

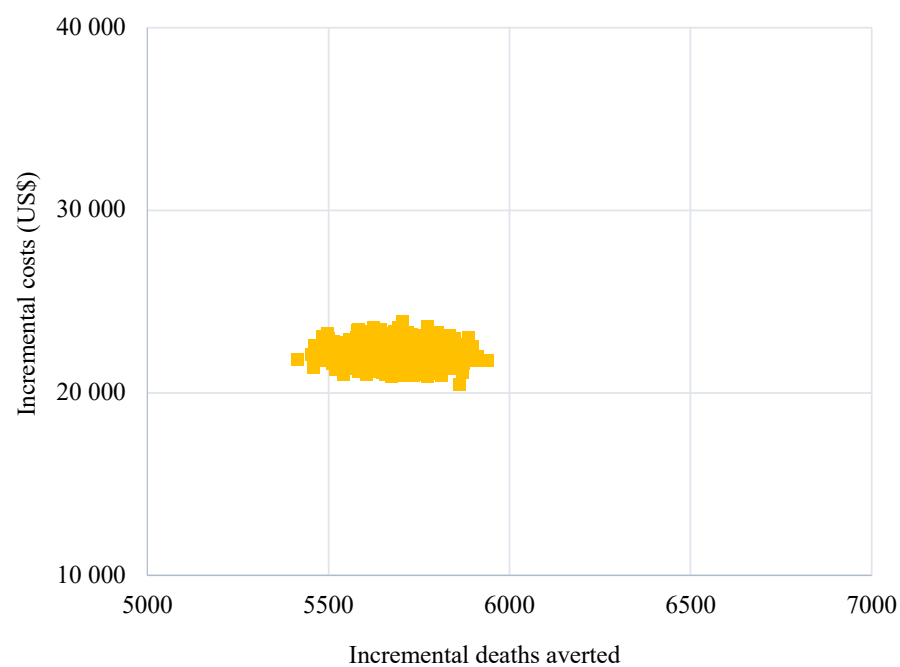

Supplement: Supplementary data [file bmjgh-2022-009553supp001.pdf]
