# Supplementary material for: Cost-effectiveness of a direct to beneficiary mobile communication programme in improving reproductive and child health outcomes in India
Source: BMJ Glob Health. 2023 Mar 23;6(Suppl 5):e009553. doi: 10.1136/bmjgh-2022-009553 (PMC10175950; doi:10.1136/bmjgh-2022-009553)
Supplement: Supplementary data [file bmjgh-2022-009553supp003.pdf]

Reflexivity statement

The demand for evidence on the cost effectiveness of Kilkari was first vocalized by the Ministry of Health and Family Welfare, as well as funders (Bill and Melinda Gates Foundation (BMGF)) and implementing partners (BBC Media Action). Kilkari is the world's largest direct to beneficiary mobile health service. Study findings provide the most robust evidence to date of the value for money of a direct to beneficiary mobile health program being implemented at scale, under real world conditions. The study research team includes researchers based in India (AB, AC, PD, OU) as well as those who have spent decades working in India (AEL, KS, DM, SC) and/or are originally from India (DM, NS). The research design and plan for implementation was germinated in India, in collaboration with local research and implementing partners. Over half of the total research budget was used to support the local research team at Oxford Policy Management-India (OPM). Members of the research team from outside of India – including collaborators at JHU and UCT—were embedded within the local research partner's implementation support structure. To this end, they attended and supported all facets of enumerator training, spent time in the field supporting data collection, and developed systems for routine analytics to identify impediments in data quality and feed these back in near real time to local field supervisors. Overarching analyses of anonymized survey data were conducted collaboratively between research partners across JHU-UCT-OPM and results fed back iteratively to program implementing partners (BBC Media Action) and funders (BMGF). This iterative approach to analytics was preceded by months of discussions across the consortium on how to define key indicators and terminology, including program reach and exposure. Consensus on these definitions across the consortium facilitated final analyses and interpretation of the results. To encourage the production of publications across the consortium, each core team member was supported to lead at least one publication (see broader supplement). Field level research team members are thanked in the acknowledgments in gratitude for their months of exhaustive efforts to implement survey tools and generate the foundational data underpinning this paper. The first author of the article is based in an LMIC institution (UCT). The second, fourth and fifth authors are early career researchers and the authorship team includes men and women; heavily tilting towards to the latter.
